# Chatgpt vs traditional pedagogy: a comparative study in urological learning

**DOI:** 10.1007/s00345-025-05654-w

**Published:** 2025-05-08

**Authors:** Alessio Digiacomo, Angelo Orsini, Rossella Cicchetti, Ludovica Spadano, Sara De Santis, Laura Di Sessa, Miriana Vitale, Marta Di Nicola, Flavia Tamborino, Martina Basconi, Riccardo De Archangelis, Gaetano Salzano, Guglielmo Dello Stritto, Peppino Lannutti, Luigi Schips, Michele Marchioni

**Affiliations:** 1 Department of Medical, Oral and Biotechnological Sciences, “G. d’Annunzio” University of Chieti-Pescara, SS Annunziata Hospital, Urology Unit, Chieti, Italy; 2 Italian Secretariat for Medical Students (SISM), Chieti, Italy; 3 Department of Medical, Oral and Biotechnological Sciences, “G. d’Annunzio” University of Chieti-Pescara, Chieti, Italy

**Keywords:** ChatGPT, Learning methods, Medical education, Pedagogy, Urology

## Abstract

**Purpose:**

Technological evolution is radically changing medical learning models. We evaluated the learning outcomes of urological concepts using ChatGPT, traditional lecture and combined approach.

**Methods:**

We conducted a randomized triple-blind study on 121 medical students with no previous formal curriculum in urology. Students were randomly divided into three study classes with different learning methods: ChatGPT, Lecture and ChatGPT + Lecture. The “adrenal glands” were randomly extracted as the subject of the lessons. Students were evaluated using a thirty-question test.

**Results:**

The evaluation test median score was higher for students who underwent ChatGPT + Lecture compared with those who had only ChatGPT (10 vs. 12, *p* = 0.007). Such differences remained statistically significant also in multivariable models adjusting according to year of course, gender and previous ChatGPT experience (estimate: 2.6, p-value = 0.002). For most of the questions (about 70%), the proportion of students correctly answering was higher in the ChatGPT + Lecture learning groups than in the other groups.

**Conclusion:**

ChatGPT loses its potential if used without a previous background. The limits of scientific reliability persist and a teacher-guided method is still essential. ChatGPT + traditional lecture gives more effective results than the single traditional lecture also allowing a better use of the chatbot.

**Supplementary Information:**

The online version contains supplementary material available at 10.1007/s00345-025-05654-w.

## Introduction

Learning and teaching methods cover a lead role in medical education and define the skills of future physicians. Medical education has been traditionally based on lectures and on a teacher who provided an already organized and predefined knowledge [1]. The rising attention for learning methods is progressively leading to their redefinition passing from an associationist concept to a cognitive one [2]; in fact, in the last years many other ways have been experimented with: team-based learning [3], simulation-based learning [4], problem-based learning [5], case-based learning [6]. In these ways, students have had an active role in the surrounding reality and learning has become an active and interactive process [1].

The technological evolution of recent years has played a central role in this radical change of conventional learning models [7]. Emerging technologies, such as e-learning, mobile-learning and Artificial Intelligence (AI), have led to the rise of heutagogy. Heutagogy is an educational approach focused on self-directed learning, where students take full responsibility for their learning journey, defining their own goals and methods [8]. Recently, AI-based tools, such as Large Language Models, have been used for their potential impact on education [9].

In this scenario, the role that ChatGPT can play in shaping medical education is highly relevant and still under evaluation [10]. Several studies have evaluated ChatGPT as a self-learning tool for medical students, primarily testing its performance on university exams or medical licensing tests [11–13].

However, none have explored its role in the teaching and learning process, either alone or combined with traditional lectures. With this in mind, we aimed to address this void by evaluating the learning outcomes of urological concepts using ChatGPT in comparison with traditional lecture and exploring the potential of combining both approaches.

## Materials and methods

We conducted a prospective, single-center, randomized, triple-blind study on a total of 121 students enrolled in the 3rd and 4th year of the Medicine and Surgery course at the University “G. d’Annunzio” with no previous formal curriculum in urology.

### Description of groups

The lists of enrolled students underwent a stratified randomization by year of the course to eliminate selection biases and obtain homogeneous samples. The students were divided by random assignment into 3 study classes composed as follows:

ChatGPT (*n* = 41): students studied the assigned topic exclusively through questions asked by themselves to ChatGPT. They autonomously acquired knowledge through self-directed exploration for a total of 90 min.

Lecture (*n* = 39): students studied the assigned topic through a 90-minute lecture using material previously prepared by the teacher.

ChatGPT + Lecture (*n* = 41): students initially used ChatGPT and then revisited the same topic through a lecture for a total time of 90 min.

Every randomization was performed using Python (version 3.11.8; Python Software Foundation, available at https://www.python.org) and the Pandas library (version 2.2.1, pandas library documentation can be found at https://pandas.pydata.org). ChatGPT 3.5 was used, allowing free access for all the students across all groups without predefined prompts or guidance, to replicate a real-life self-directed learning experience. This ensured that all students had the same level of freedom in formulating their questions, maintaining consistency across groups.

### Sample size Estimation

The number of students to be included within the three groups was evaluated using G*Power (version 3.1). Sample size estimation was performed a priori hypothesizing a large effect size (Cohen’s effect size 0.4) with 5% acceptable alpha error and a desired power of 95%. The resulting total sample size was 102 students. Since we expected that a proportion of students might not participate in the didactic activities, even if they were registered, the total number of students that could be potentially enrolled was increased by 20%.

### Topic and questions extraction

The choice of the topic was made through random draw from the book “Campbell-Walsh Urology (Twelfth Edition)“ [14]. The “adrenal glands” were extracted as the subject of the lessons. The teacher, a medical doctor and associate professor of urology at our University, was informed about the topic only 2 weeks before the lesson to prepare a presentation on the subject. ChatGPT 4.0 was used by the teacher and attempted to create images and multimedia material to be used in the lecture. The specific prompt given was: *“Generate detailed and anatomically accurate illustrations for a university-level lecture on the adrenal glands*,* depicting their anatomy*,* vascularization*,* function*,* hormone-related pathologies*,* neoplastic diseases*,* and robotic adrenal surgery*,* following standard medical references for accuracy and clarity”*. The teacher knew nothing about the evaluation test questions. The students were blind to the topic until the beginning of the different learning modalities to avoid the acquisition of previous notions.

At the end of the learning phase, students were evaluated using a thirty-question test taken from the book “Campbell-Walsh-Wein Urology Twelfth Edition Review, third edition” [15] related to the topic (Supplementary material). Selected questions belonged to six main topics: Anatomy and Diagnostics, Physiology, Hormonal Pathologies, Neoplastic Pathologies, Surgery and Clinical Cases. Correct answers scored one, incorrect/unanswered answers scored zero marks. The test was carried out simultaneously by the 3 classes in 60 min.

### Data analysis

Descriptive statistics relied on absolute and relative frequencies (%) for qualitative data and on median and interquartile ranges (IQR) for quantitative variables. Differences between groups in medians were tested by the Wilcoxon test and post-hoc analysis was conducted with the Bonferroni adjustment method; differences in proportions were tested by the Chi-square test. Linear regression models tested differences between classes in the overall cohort and within subgroups defined according to gender, year of study and previous experience with ChatGPT. (Supplementary material).

Statistical analyses were performed by a statistician blinded to the nature of the groups using R Statistical Software (version 4.0.0; R Foundation for Statistical Computing, Vienna, Austria). A p-value less than 0.05 was defined as statistically significant.

## Results

### Baseline characteristics

**Fig. 1:**
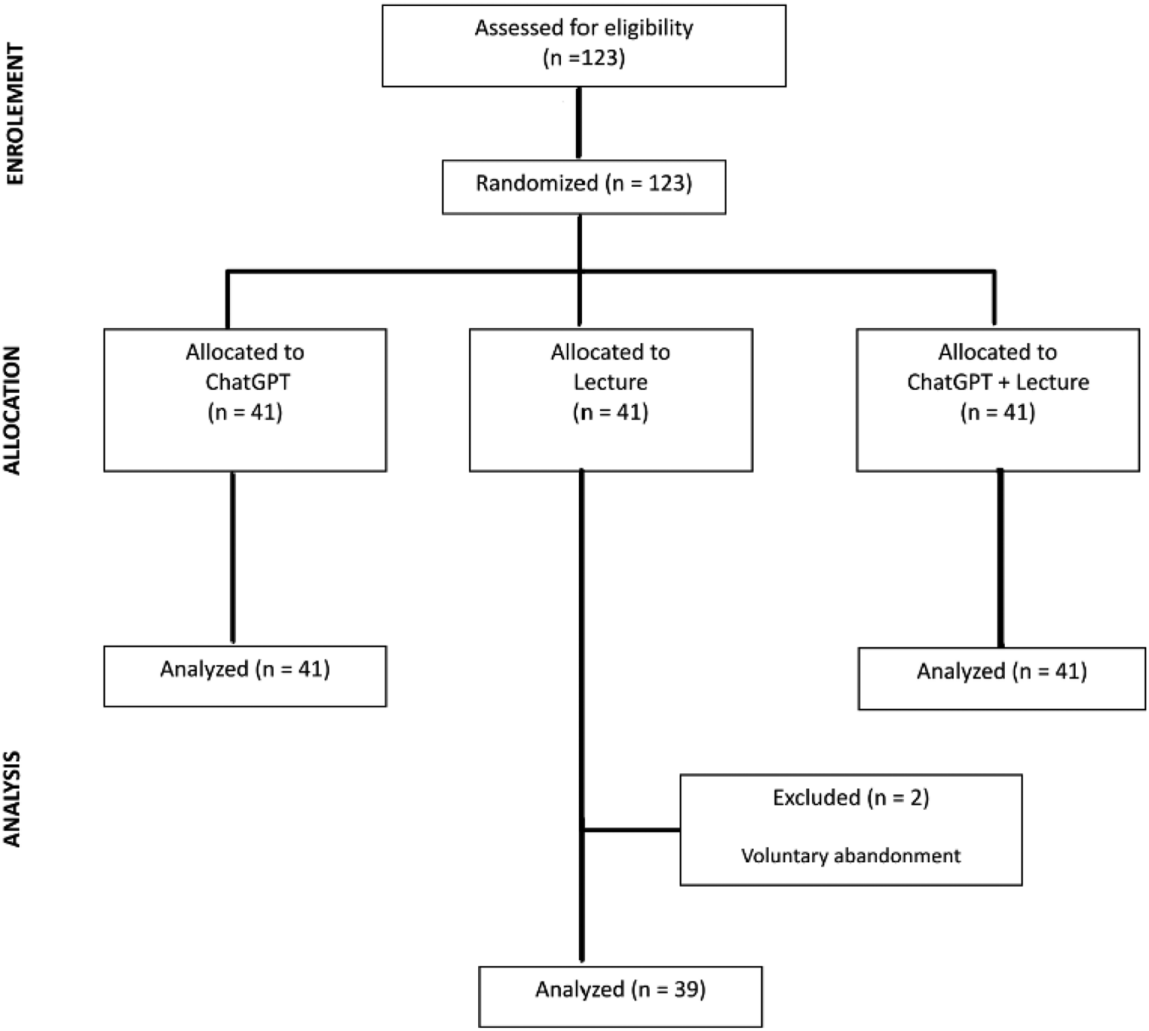
CONSORT flow diagram of student enrollment, group allocation and analysis

Overall, 121 students were enrolled and randomized in three classes (Fig. 1). Students median age was 23.8 years (IQR 22–25 years), mostly female 75/121 (62%), from the 3rd year course 76/121 (63%) and with no experience using ChatGPT for studying 61/121 (50%).

#### Effect of ChatGPT on students’ evaluation test score

**Fig. 2:**
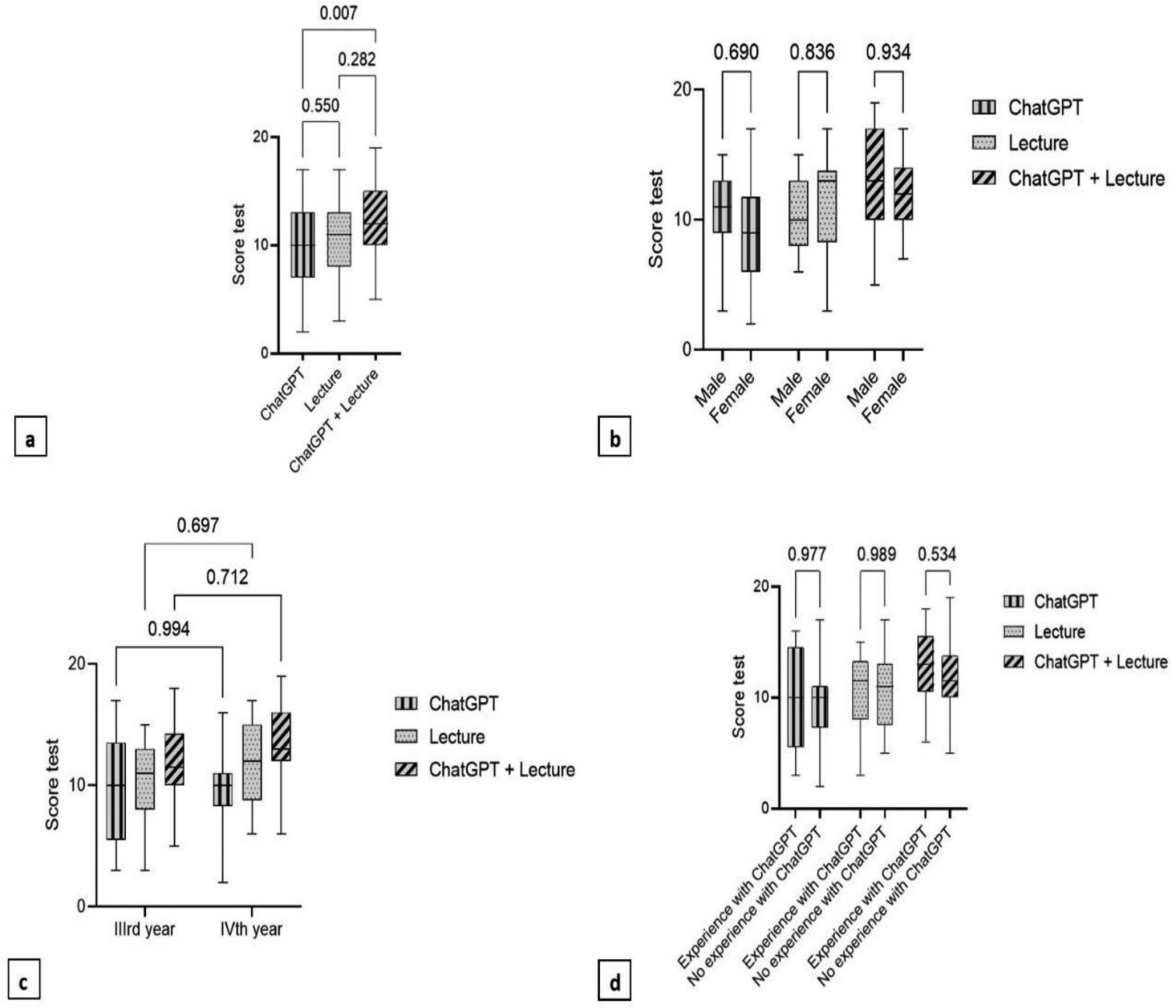
Box plots showing: **a**) evaluation test median score for each class (named according to the learning method: ChatGPT, Lecture, ChatGPT + Lecture). No statistically significant differences were found according to **b**) gender, **c**) year of study or **d**) previous experience with ChatGPT. All p-values from post-hoc analyses were adjusted according to Bonferroni method

Median score was higher for those who underwent ChatGPT + Lecture compared to those who had only ChatGPT (10 vs. 12, *p* = 0.007; Fig. 2a). No statistically significant differences were found according to gender (Fig. 2b), year of study (Fig. 2c) or previous experience with ChatGPT (Fig. 2d).

Univariable and multivariable linear regression models showed better performances for those undergoing ChatGPT + Lecture than ChatGPT alone. Results remained virtually the same also in subgroups defined according to gender, year of course and experience with ChatGPT (Table 1).

**Table 1:** Subgroup analysis of learning outcomes: univariable and multivariable regression models

Classes	Estimate (± Standard error)	*p*-value
**Univariable model**		
ChatGPT	Reference	
Lecture	1.2 (± 0.8)	0.130
ChatGPT + Lecture	2.7 (± 0.8)	< 0.001
**Multivariable model (adjusted according to gender**,** year of course**,** experience with ChatGPT)**		
ChatGPT	Reference	
Lecture	1.0 (± 0.8)	0.218
ChatGPT + Lecture	2.6 (± 0.8)	0.002
**Male**		
ChatGPT	Reference	
Lecture	− 0.2 (± 1.3)	0.893
ChatGPT + Lecture	2.1 (± 1.3)	0.110
**Female**		
ChatGPT	Reference	
Lecture	1.9 (± 1.0)	0.052
ChatGPT + Lecture	2.9 (± 1.0)	0.004
**3rd year study**		
ChatGPT	Reference	
Lecture	0.9 (± 1.0)	0.375
ChatGPT + Lecture	2.4 (± 1.0)	0.018
**4th year study**		
ChatGPT	Reference	
Lecture	1.8 (± 1.3)	0.183
ChatGPT + Lecture	3.2 (± 1.3)	0.017
**ChatGPT experience**		
ChatGPT	Reference	
Lecture	0.9 (± 1.2)	0.458
ChatGPT + Lecture	3.0 (± 1.2)	0.017
**No previous ChatGPT experience**		
ChatGPT	Reference	
Lecture	1.4 (± 1.2)	0.262
ChatGPT + Lecture	2.1 (± 1.1)	0.049

**Fig. 3:**
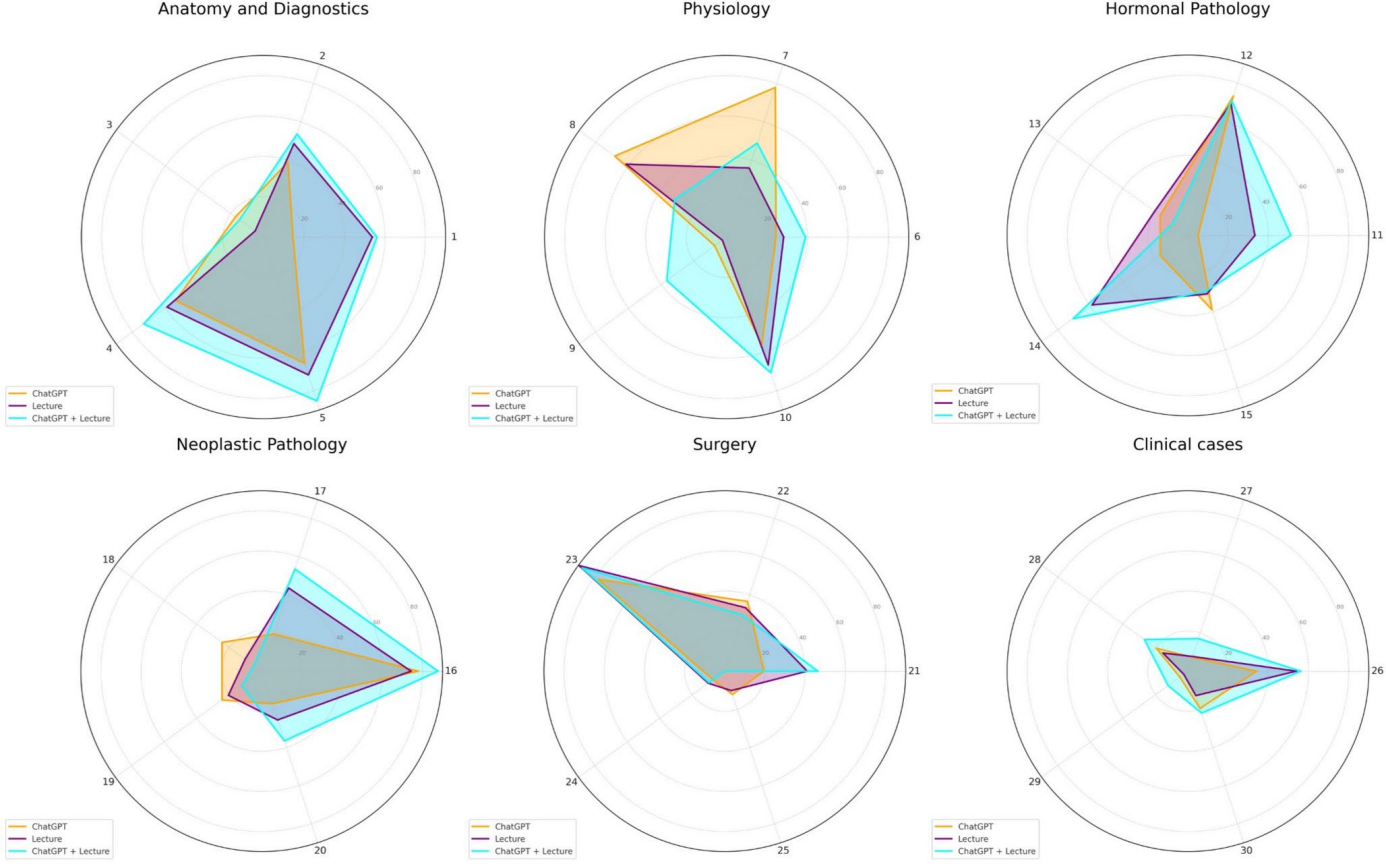
Radar graphs showing the distribution of correct answers across main topics for each class

For most of the questions (about 70%), the proportion of students correctly answering was higher in the ChatGPT + Lecture learning groups than in the other groups (Fig. 3 and Supplementary material).

#### Use of ChatGPT for educational resource production

**Fig. 4:**
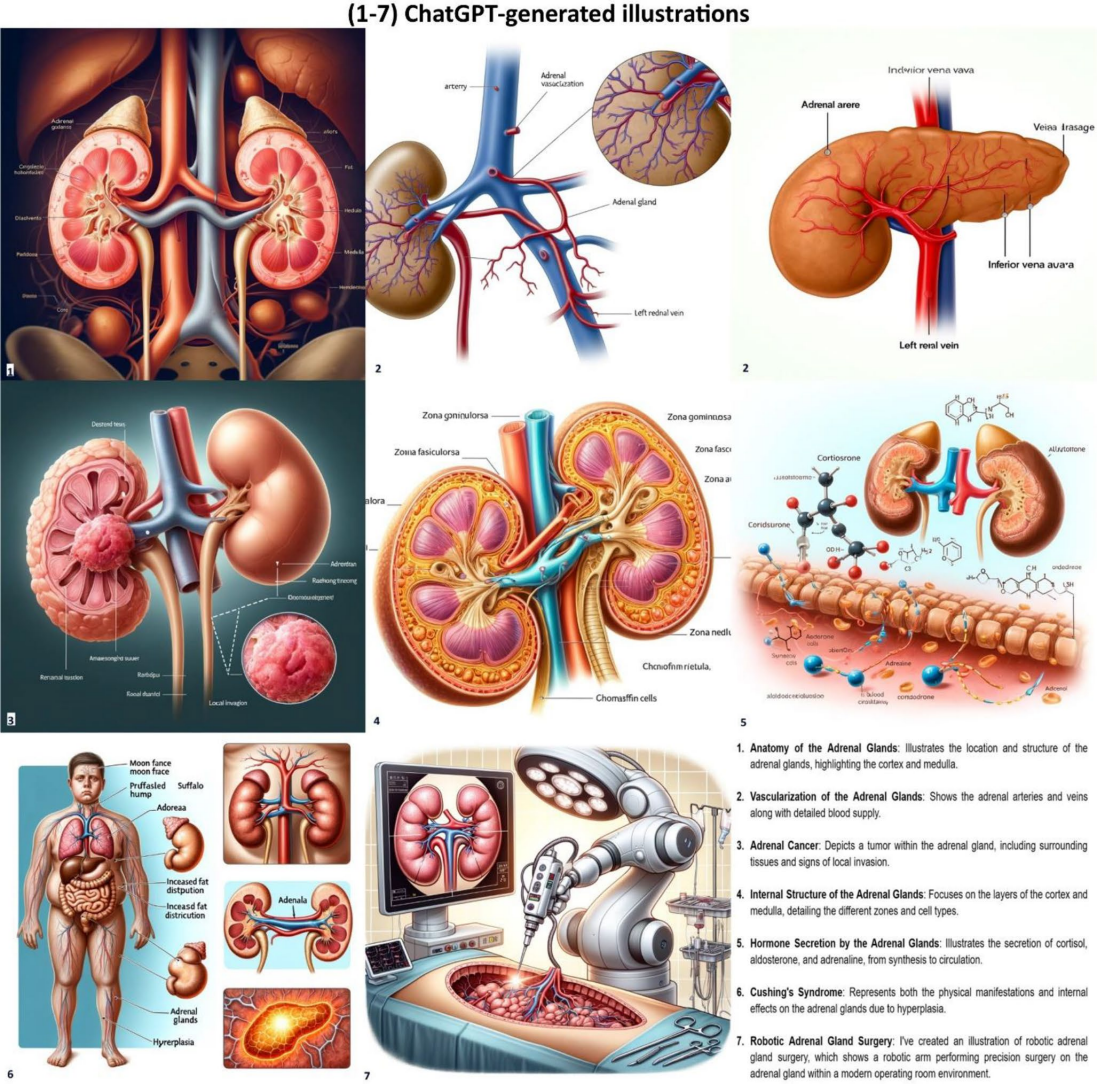
All images were generated using ChatGPT-4.0. This attempt revealed a deep lack of scientific accuracy in the illustrations, despite ChatGPT describing them as scientifically accurate (as shown in the last image)

ChatGPT 4.0 was used by the teacher to make images and multimedia material for the lecture. This attempt revealed a significant lack of scientific accuracy in the images (Fig. 4), which lacked validity and contained texts with grammatical errors or nonsensical content.

## Discussion

This study analyzes learning outcomes of urological concepts using ChatGPT in comparison with traditional lecture and explores the potential of combining both approaches. Our study showed that the use of a combination of ChatGPT and traditional learning tools warrant a better performance. Our results are of great interest since this is the first formal study testing this hypothesis.

Many studies tested ChatGPT performance using university exam and medical licensing test questions, to analyze the accuracy of the answers [11–13]. Kung et al. [12] found that ChatGPT answered United States Medical Licensing Exam (USMLE) questions with good accuracy (> 50%) and high agreement. Gilson et al. [16] also tested ChatGPT using questions from the USMLE exam. It achieved a passing score, providing logical and informative responses. Conversely, Fijačko et al. [17], showed that ChatGPT failed the Basic Life Support (BLS) and Advanced Cardiovascular Life Support (ACLS) exams, despite its scientifically relevant answers. In Eysenbach’s study [18], the potential and limits of ChatGPT are empirically and broadly tested through conversation with the tool. In several points the user must direct and correct the chatbot with suggestions about the topics, the correct way to conduct an anamnesis, or provide information to a patient within the simulations carried out. Clear limitations are still those expressed in the interview by ChatGPT itself: the lack of direct generation of diagrams and animations, the absence of critical analysis skills, the lack of access to all scientific articles, the need for professional medical supervision for critical content analysis.

In addition, we addressed all the other different gaps that remain in the ChatGPT usability as a self-learning tool from the neophyte medical students’ perspective. Even if the studies show ChatGPT performance supporting its usefulness as a self-learning tool, we obtained lower test evaluation scores with the use of ChatGPT alone compared to the lecture alone. Despite the potential of ChatGPT in self-directed study and improving critical clinical thinking, we demonstrate a higher efficacy of traditional lecture compared to the standalone use of ChatGPT in agreement with the critical analysis carried out by Qu et al. [19]. Recently, Şahin et al. [20] conducted a comparative analysis assessing the accuracy of five chatbots (GPT-4o, Copilot Pro, Gemini Advanced, Claude 3.5, and Sonar Huge) in answering questions from the European Board of Urology In-Service Assessment (ISA), one of the most reliable tools for evaluating urological theoretical knowledge. The results show that chatbots perform better on purely theoretical questions than on those requiring clinical reasoning or interpretation. Of particular relevance is the methodological distinction introduced between factual knowledge and critical clinical thinking, which reveals that current Language Models still struggle with context-based reasoning. These findings reinforce a key point also highlighted in our study: while ChatGPT shows promising potential as a learning tool, its educational effectiveness remains limited in the absence of structured guidance or a solid theoretical foundation. From a pedagogical point of view, Gayef et al. [21] explain how better results are related to intrinsic student motivation and independent learning methods (e.g. ChatGPT). We found higher test scores in students who used ChatGPT + traditional lecture compared to the ones who attended lecture alone. As in the article by Gayef et al., this confirms the usefulness of an independent study method. Analyses show a gradual increase in the evaluation test median scores moving from the use of ChatGPT to lecture only, and finally to the blended use of ChatGPT + lecture. Gayef et al. [21] highlight the chatbot’s ability to fabricate scientific references, a phenomenon known as “hallucination”, where AI-generated responses contain false information [22]. Similarly, when the teacher used ChatGPT 4.0 to create lecture images, the results lacked medical validity, with texts full of grammatical errors or meaningless content. The studies by Şahin et al. [23] and Malak et al. [24] compare multiple chatbots, confirming variability in reliability, quality, and the need for prior knowledge or supervision. They also highlight differences in accessibility and comprehensibility, suggesting further research on chatbot roles in self-learning and the impact of accuracy and clarity. Although our study is the first to examine ChatGPT’s impact in a structured learning process, it has limitations: a short learning phase and the lack of a repeated evaluation test after a longer period to confirm the effectiveness of different learning approaches or to identify potential variations in outcomes.

## Conclusions

Our study confirms ChatGPT’s high potential in learning for both educators and learners but highlights its limitations when used without prior knowledge. Key considerations remain persisting limits in scientific reliability and the essential role of teacher guidance. Blended learning (ChatGPT + lecture) is both possible and desirable, as it yields more effective results than the traditional method alone and, moreover, allows for a critical and conscious use of the tool.

## Electronic supplementary material

Below is the link to the electronic supplementary material.

Supplementary Material 1

## Data Availability

Data is provided within the manuscript or supplementary information files.The authors are available to provide the codes and additional materials used in the study upon request from the reviewers.

## Code availability

The authors are available to provide the codes and materials used in the study upon request from the reviewers.

## References

[1] Zhang S, Zhu D, Wang X, Liu T, Wang L, Fan X, Gong H (2024) Effects of six teaching strategies on medical students: protocol for a systematic review and network meta-analysis. BMJ Open 14(1):e079716. 10.1136/bmjopen-2023-079716PMID: 38296281; PMCID: PMC10828868 38296281 10.1136/bmjopen-2023-079716 PMC10828868

[2] Blaschke LM (2012) Heutagogy and lifelong learning: A review of heutagogical practice and self-determined learning. Int Rev Res Open Distrib Learn 13(1):56–71. 10.19173/irrodl.v13i1.1076

[3] Parmelee D, Michaelsen LK, Cook S, Hudes PD (2012) Team-based learning: a practical guide: AMEE guide no. 65. Med Teach. 34(5):e275-87. 10.3109/0142159X.2012.651179. Epub 2012 Apr 4. PMID: 22471941 10.3109/0142159X.2012.651179 22471941

[4] Issenberg SB, McGaghie WC, Petrusa ER, Lee Gordon D, Scalese RJ (2005) Features and uses of high-fidelity medical simulations that lead to effective learning: a BEME systematic review. Med Teach. 27(1):10–28. 10.1080/01421590500046924. PMID: 16147767 10.1080/01421590500046924 16147767

[5] Kwan CY (2019) A thorny path: the developmental course of problem-based learning for health sciences education in Asia. Adv Health Sci Educ Theory Pract 24(5):893–901. 10.1007/s10459-019-09920-6 Epub 2019 Oct 22. PMID: 31641943 31641943 10.1007/s10459-019-09920-6

[6] McLean SF (2016) Case-Based Learning and its Application in Medical and Health-Care Fields: A Review of Worldwide Literature. J Med Educ Curric Dev 3. 10.4137/JMECD.S20377 PMID: 29349306; PMCID: PMC5736264: JMECD.S20377 10.4137/JMECD.S20377 PMC5736264 29349306

[7] Muttappallymyalil J, Mendis S, John LJ, Shanthakumari N, Sreedharan J, Shaikh RB (2016) Evolution of technology in teaching: blackboard and beyond in medical education. Nepal J Epidemiol 6(3):588–592. 10.3126/nje.v6i3.15870 PMID: 27822404; PMCID: PMC5082488 27822404 10.3126/nje.v6i3.15870 PMC5082488

[8] Bansal A, Jain S, Sharma L, Sharma N, Jain C, Madaan M (2020) Students’ perception regarding pedagogy, andragogy, and heutagogy as teaching-learning methods in undergraduate medical education. J Educ Health Promot 9:301. 10.4103/jehp.jehp_221_20 PMID: 33426105; PMCID: PMC7774633 33426105 10.4103/jehp.jehp_221_20 PMC7774633

[9] 2023 EDUCAUSE Horizon Report| Teaching and Learning Edition| EDUCAUSE Library. https://library.educause.edu/resources/2023/5/2023-educause-horizon-report-teaching-and-learning-edition

[10] Vignesh R, Pradeep P, Balakrishnan P (2023) A Tête-à-tête with ChatGPT on the impact of artificial intelligence in medical education. Med J Malaysia 78:547–549 37518931

[11] Zalzal HG, Cheng J, Shah RK (2023) Evaluating the current ability of ChatGPT to assist in professional otolaryngology education. OTO Open 7:e94 38020045 10.1002/oto2.94 PMC10663981

[12] Kung TH et al (2023) Performance of ChatGPT on USMLE: potential for AI-assisted medical education using large Language models. PLOS Digit Health 2:e0000198 36812645 10.1371/journal.pdig.0000198 PMC9931230

[13] Surapaneni KM et al (2024) Evaluating ChatGPT as a self-learning tool in medical biochemistry: A performance assessment in undergraduate medical university examination. Biochem Mol Biol Educ 52:237–248 38112255 10.1002/bmb.21808

[14] Campbell Walsh Wein Urology - 9780323546423| Elsevier Health. MEA Elsevier Health https://www.mea.elsevierhealth.com/campbell-walsh-wein-urology-9780323546423.html

[15] Partin AW, Peters CA, Kavoussi LR, Dmochowski RR, Wein AJ (2020) Campbell-Walsh-Wein Urology Twelfth Edition Review (3rd ed.). Elsevier

[16] Gilson A et al (2023) How does ChatGPT perform on the united States medical licensing examination (USMLE)? The implications of large Language models for medical education and knowledge assessment. JMIR Med Educ 9:e45312 36753318 10.2196/45312 PMC9947764

[17] Fijačko N, Gosak L, Štiglic G, Picard CT (2023) John Douma, M. Can ChatGPT pass the life support exams without entering the American heart association course? Resuscitation 185:109732 36775020 10.1016/j.resuscitation.2023.109732

[18] Eysenbach G (2023) The role of ChatGPT, generative Language models, and artificial intelligence in medical education: A conversation with ChatGPT and a call for papers. JMIR Med Educ 9:e46885 36863937 10.2196/46885 PMC10028514

[19] Qu X, Yang J, Chen T, Zhang W (2023) [Reflections on the implications of the developments in ChatGPT for changes in medical education models]. Sichuan Da Xue Xue Bao Yi Xue Ban 54(5):937–940 Chinese. doi: 10.12182/20231360302. PMID: 37866949; PMCID: PMC10579070 37866949 10.12182/20231360302 PMC10579070

[20] Şahin MF, Doğan Ç, Topkaç EC, Şeramet S, Tuncer FB, Yazıcı CM (2025) Which current chatbot is more competent in urological theoretical knowledge? A comparative analysis by the European board of urology in-service assessment. World J Urol 43(1):116 39932577 10.1007/s00345-025-05499-3 PMC11813998

[21] Gayef A, Çaylan A, Temiz SA (2023) Learning styles of medical students and related factors. BMC Med Educ 23(1):282. 10.1186/s12909-023-04267-4PMID: 37098595; PMCID: PMC10131376 37098595 10.1186/s12909-023-04267-4 PMC10131376

[22] Maleki N, Padmanabhan B, Dutta K (2024) AI hallucinations: A misnomer worth clarifying. arXiv:2401.06796v1 [cs.CL]

[23] Şahin MF, Topkaç EC, Doğan Ç, Şeramet S, Özcan R, Akgül M, Yazıcı CM (2024) Still using only ChatGPT? The comparison of five different artificial intelligence chatbots’ answers to the most common questions about kidney stones. J Endourol 38(11):1172–1177 39212674 10.1089/end.2024.0474

[24] Malak A, Şahin MF (2024) How useful are current chatbots regarding urology patient information?? Comparison of the ten most popular chatbots’ responses about female urinary incontinence. J Med Syst 48(1):102 39535651 10.1007/s10916-024-02125-4

