# Supplementary material for: Chatgpt vs traditional pedagogy: a comparative study in urological learning
Source: World J Urol. 2025 May 8;43(1):286. doi: 10.1007/s00345-025-05654-w (PMC12062111; doi:10.1007/s00345-025-05654-w)
Supplement: Supplementary file 1 — Supplementary Material 1 [file 345_2025_5654_MOESM1_ESM.docx]

**World Journal of Urology**

**CHATGPT VS TRADITIONAL** **PEDAGOGY: A COMPARATIVE STUDY IN UROLOGICAL LEARNING**

Alessio Digiacomo^1^, Angelo Orsini^1^, Rossella Cicchetti^1^, Ludovica Spadano^2^, Sara De Santis^2^, Laura Di Sessa^2^, Miriana Vitale^2^, Marta Di Nicola^3^, Flavia Tamborino^1^, Martina Basconi^1^, Riccardo De Archangelis^1^, Gaetano Salzano^1^, Guglielmo Dello Stritto^1^, Peppino Lannutti^1^, Luigi Schips^1^, Michele Marchioni^1^

^1^ Department of Medical, Oral and Biotechnological Sciences, “G. d’Annunzio” University of Chieti-Pescara, Urology Unit, SS Annunziata Hospital, Chieti, Italy

^2^ Italian Secretariat for Medical Students (SISM) - Chieti, Italy

^3^ Department of Medical, Oral and Biotechnological Sciences, “G. d’Annunzio” University of Chieti-Pescara, Chieti, Italy

**Corresponding author:**

Alessio Digiacomo, MD

“G. d’Annunzio” University, Chieti, Italy

Phone: +39 3279975054

[ale.digiacomo.uro@gmail.com](mailto:ale.digiacomo.uro@gmail.com)

ORCID iD: 0009-0000-9312-2266

**Supplementary material**

This supplementary material provides the thirty-question test showing for each learning method the percentage of students who gave the correct answer and the corresponding p-value.

| **Anatomy and Diagnostics** | | |
| --- | --- | --- |
| **QUESTION 1.** In cases of renal ectopia, the ipsilateral adrenal gland is typically: | | |
| 1. absent. 2. **found in its normal anatomic position in the upper retroperitoneum.** 3. found in association with the contralateral adrenal gland. 4. found closely applied to the superior pole of the ectopic kidney. 5. found closely associated with the ipsilateral renal artery. | ChatGPT: 6/41 students (14.6%) | p < 0.001 |
|  | Lecture: 21/39 students (53.8%) |  |
|  | ChatGPT + Lecture: 23/41 students  (56.1%) |  |
|  |  |  |
| **QUESTION 2**. Which of the following statements is TRUE? | | |
| 1. Ultrasound imaging cannot differentiate between solid and cystic masses of the adrenal gland. 2. Contrast resolution of magnetic resonance imaging (MRI) is inferior to that of computed tomography (CT) in enabling differentiation of adrenal masses. 3. **CT is the most widely used modality for imaging the adrenal glands.** 4. Normal adrenal tissue has a density of greater than 10   Hounsfield units on non contrast CT imaging.   1. MRI, via T1-weighted and T2-weighted images, can provide functional data about adrenal masses. | ChatGPT: 16/41 students (39%) | p = 0.40 |
|  | Lecture: 19/39 students (48.7%) |  |
|  | ChatGPT + Lecture: 22/41 students  (53.7%) |  |
|  |  |  |
| **QUESTION 3.** What subtype is assigned to patients with a von Hippel-Lindau (VHL) mutation and a history of pheochromocytoma but no other stigmata of the VHL syndrome? | | |
| 1. Type 1 2. Type 2A 3. Type 2B 4. **Type 2C** 5. None of the above | ChatGPT: 7/41 students (17.1%) | p = 0.23 |
|  | Lecture: 2/39 students (5.1%) |  |
|  | ChatGPT + Lecture: 6/41 students  (14.6%) |  |
|  |  |  |
| **QUESTION 4.** In cases of unilateral renal agenesis, the ipsilateral adrenal gland is commonly: | | |
| 1. absent. 2. **found in its normal anatomic position in the upper retroperitoneum.** 3. found in association with the contralateral adrenal gland. 4. found just inside the ipsilateral internal inguinal ring. 5. found in an ectopic, intrathoracic location. | ChatGPT: 22/41 students (53.6%) | p = 0.17 |
|  | Lecture: 23/39 students (59%) |  |
|  | ChatGPT + Lecture: 30/41 students  (73.2%) |  |
|  |  |  |
| **QUESTION 5.** Which of the following statements is (are) TRUE? | | |
| 1. The weight of each of the glands is approximately 10 g. 2. The adrenal glands are in close proximity to the crus of the diaphragm. 3. The right gland is crescentic in shape. 4. The left adrenal gland lies adjacent to the splenic vessels. 5. **Both b and d.** | ChatGPT: 27/41 students (65.8%) | p = 0.11 |
|  | Lecture: 28/39 students (71.8%) |  |
|  | ChatGPT + Lecture: 35/41 students  (85.4%) |  |
|  |  |  |

| **Physiology** | | |
| --- | --- | --- |
| **QUESTION 6.** Elevated aldosterone in patients with familial hyperaldosteronism type I is mediated by: | | |
| 1. renin. 2. sodium. 3. angiotensin II. 4. cortisol. 5. **ACTH.** | ChatGPT: 10/41 students (24.4%) | p = 0.32 |
|  | Lecture: 11/39 students (28.2%) |  |
|  | ChatGPT + Lecture: 16/41 students  (39%) |  |
|  |  |  |
| **QUESTION 7**. Presence of the phenylethanolamine-N-methyltransferase (PNMT) enzyme in the adrenal medulla is significant because: | | |
| 1. the enzyme catalyzes degradation of catecholamines to metanephrines. 2. the enzyme catalyzes conversion of catecholamines to vanillylmandelic acid (VMA). 3. the enzyme converts tyrosine to dopamine. 4. **the enzyme catalyzes the conversion of norepinephrine to epinephrine.** 5. all of the above. | ChatGPT: 32/41 students (78%) | p = 0.0005 |
|  | Lecture: 14/39 students (36%) |  |
|  | ChatGPT + Lecture: 20/41 students  (49%) |  |
|  |  |  |
| **QUESTION 8.** The most common hormone secreted by adrenocortical carcinoma is: | | |
| 1. aldosterone. 2. testosterone. 3. dehydroepiandrosterone (DHEA). 4. **cortisol.** 5. androstenedione. | ChatGPT: 28/41 students (68.3%) | p = 0.002 |
|  | Lecture: 24/39 students (61.5%) |  |
|  | ChatGPT + Lecture: 13/41 students  (31.7%) |  |
|  |  |  |
| **QUESTION 9.** The most abundant product of the adrenal cortex is: | | |
| 1. mineralocorticoids. 2. glucocorticoids. 3. **adrenal androgens.** 4. catecholamines. 5. adrenocorticotropic hormone (ACTH). | ChatGPT: 3/41 students (7.3%) | p = 0.0001 |
|  | Lecture: 1/39 students (2.6%) |  |
|  | ChatGPT + Lecture: 15/41 students  (36.6%) |  |
|  |  |  |
| **QUESTION 10.** Metanephrines: | | |
| 1. refers to the term used for catecholamines and their byproducts. 2. **refers to the combined term for methylated metabolites of norepinephrine (normetanephrine) and epinephrine**   **(metanephrine).**   1. refers to precursors to normetanephrines. 2. are rarely helpful in establishing a diagnosis of   pheochromocytoma.   1. refers to the term used to describe epinephrine and norepinephrine in the context of pheochromocytoma symptomatology. | ChatGPT: 23/41 students (56.1%) | p = 0.36 |
|  | Lecture: 26/39 students (66.6%) |  |
|  | ChatGPT + Lecture: 29/41 students  (70.7%) |  |
|  |  |  |

| **Hormonal Pathology** | | |
| --- | --- | --- |
| **QUESTION 11.** What percentage of patients presenting with primary aldosteronism are hypokalemic? | | |
| 1. 5% to 12% 2. **9% to 37%** 3. 33% to 50% 4. 55% to 75% 5. 63% to 91% | ChatGPT: 2/41 students (5%) | p = 0.0001 |
|  | Lecture: 13/39 students (33.3%) |  |
|  | ChatGPT + Lecture: 21/41 students  (51.2%) |  |
|  |  |  |
| **QUESTION 12**. What common urologic ailment can be found in up to 50% of patients with Cushing syndrome? | | |
| 1. Testicular cancer 2. Torsion of the appendix testis 3. **Urolithiasis** 4. Fournier gangrene 5. Stress urinary incontinence | ChatGPT: 30/41 students (73.2%) | p = 0.92 |
|  | Lecture: 27/39 students (69.2%) |  |
|  | ChatGPT + Lecture: 29/41 students  (70.7%) |  |
|  |  |  |
| **QUESTION 13.** The most common cause of Cushing syndrome (exclusive of exogenous steroid intake) is: | | |
| 1. **Cushing disease.** 2. a cortisol-producing adrenal adenoma. 3. ectopic ACTH production by a lung malignancy. 4. an adrenal carcinoma. 5. a pheochromocytoma. | ChatGPT: 7/41 students (17.1%) | p = 0.39 |
|  | Lecture: 8/39 students (20.5%) |  |
|  | ChatGPT + Lecture: 4/41 students  (9.7%) |  |
|  |  |  |
| **QUESTION 14. A 50% false-positive rate can be seen during low-dose dexamethasone suppression testing in:** | | |
| 1. men with testicular cancer. 2. **women taking oral contraceptives.** 3. men with history of orchiopexy. 4. patients with brain malignancy. 5. patients with pheochromocytoma. | ChatGPT: 7/41 students (17.1%) | p = 0.0001 |
|  | Lecture: 23/39 students (59%) |  |
|  | ChatGPT + Lecture: 29/41 students  (70.7%) |  |
|  |  |  |
| **QUESTION 15.** How does one perform a low-dose dexamethasone suppression test (LDDST)? | | |
| 1. Admit the patient and measure serum cortisol levels every 6hours while the patient is on a dexamethasone drip 2. Measure the patient’s saliva cortisol level at midnight 3. Obtain a 24-hour urine cortisol measurement after the patient receives 1 mg of dexamethasone with the first void 4. Have the patient take 10 mg of dexamethasone at 11 PM and measure urinary cortisol the next morning 5. **Have the patient take 1 mg of dexamethasone at 11 PM and measure serum cortisol the next morning** | ChatGPT: 16/41 students (39%) | p = 0.60 |
|  | Lecture: 12/39 students (30.7%) |  |
|  | ChatGPT + Lecture: 12/41 students  (29.3%) |  |
|  |  |  |

| **Neoplastic Pathology** | |  |
| --- | --- | --- |
| **QUESTION 16.** In the treatment of pathologically localized adrenocortical carcinoma: | |  |
| 1. adjuvant radiation therapy decreases systemic progression. 2. **complete surgical resection offers the best chance of cure.** 3. increased Ki-67 expression has been associated with improved survival. 4. the tumor’s functional status is an independent predictor of survival. 5. adjuvant therapy with mitotane has no proven benefit. | ChatGPT: 32/41 students (78%) | p = 0.29 |
|  | Lecture: 29/39 students (74.3%) |  |
|  | ChatGPT + Lecture: 36/41 students  (87.8%) |  |
|  |  |  |
| **QUESTION 17**. Adrenocortical carcinoma in children: | |  |
| 1. **has a more favorable 5-year survival rate compared with adults.** 2. uses the same pathologic staging system as adults. 3. is rarely associated with virilization. 4. has equal female and male incidence in children older than   10 years.  e. is frequently metastatic to the central nervous system. | ChatGPT: 8/41 students (19.5%) | p = 0.005 |
|  | Lecture: 17/39 students (43.6%) |  |
|  | ChatGPT + Lecture: 22/41 students  (53.6%) |  |
|  |  |  |
| **QUESTION 18.** What genetic abnormality is strongly linked with malignant pheochromocytoma? | |  |
| 1. RET mutation 2. VHL mutation 3. **SDHB mutation** 4. SDHD mutation 5. All of the above | ChatGPT: 10/41 students (24.4%) | p = 0.02 |
|  | Lecture: 4/39 students (10.2%) |  |
|  | ChatGPT + Lecture: 2/41 students  (4.8%) |  |
|  |  |  |
| **QUESTION 19. All of the following lesions can be extra-adrenal EXCEPT:** | |  |
| 1. myelolipoma. 2. ganglioneuroma. 3. **aldosteronoma.** 4. pheochromocytoma. 5. oncocytoma. | ChatGPT: 10/41 students (24.4%) | p = 0.35 |
|  | Lecture: 8/39 students (20.5%) |  |
|  | ChatGPT + Lecture: 5/41 students  (12.2%) |  |
|  |  |  |
| **QUESTION 20.** What is the most common subtype of primary aldosteronism? | |  |
| 1. **Idiopathic hyperplasia** 2. Aldosterone-producing adenoma 3. Unilateral adrenal hyperplasia 4. Familial hyperaldosteronism type I 5. Adrenocortical carcinoma | ChatGPT: 7/41 students (17.1%) | p = 0.13 |
|  | Lecture: 10/39 students (25.6%) |  |
|  | ChatGPT + Lecture: 15/41 students  (36.6%) |  |
|  |  |  |

| **Surgery** | | |
| --- | --- | --- |
| **QUESTION 21.** Which of the following is not an advantage of retroperitoneal adrenalectomy when compared to transperitoneal approach? | | |
| 1. Reducing the risk of injury to bowels and abdominal organs 2. **Larger working space and clearer anatomical landmarks** 3. Lower incidence of hemodynamic and respiratory morbidities 4. Usually the approach of choice in patients with multiple previous abdominal surgeries 5. All of the above are advantages of the retroperitoneal approach over the transperitoneal approach | ChatGPT: 8/41 students (19.5%) | p = 0.02 |
|  | Lecture: 16/39 students (41%) |  |
|  | ChatGPT + Lecture: 19/41 students  (46.3%) |  |
|  |  |  |
| **QUESTION 22**. Which of the following is NOT a possible approach to the left adrenal gland in the anterior transabdominal approach? | | |
| 1. Through the gastrocolic ligament 2. Through the lienorenal ligament 3. Through the transverse mesocolon 4. Through the lesser omentum 5. **All the above are possible approaches** | ChatGPT: 15/41 students (36.6%) | p = 0.77 |
|  | Lecture: 13/39 students (33.3%) |  |
|  | ChatGPT + Lecture: 12/41 students  (29.3%) |  |
|  |  |  |
| **QUESTION 23.** The adrenal gland should be resected whenever one performs a radical nephrectomy. | | |
| 1. True 2. **False** | ChatGPT: 32/41 students (78%) | p = 0.20 |
|  | Lecture: 35/39 students (89.7%) |  |
|  | ChatGPT + Lecture: 36/41 students  (87.8%) |  |
|  |  |  |
| **QUESTION 24. The lumbodorsal posterior approach to open adrenalectomy is not ideal in any of the following circumstances EXCEPT:** | | |
| 1. large adrenal tumors. 2. **bilateral adrenal hyperplasia.** 3. bilateral small adrenocortical carcinoma. 4. patients with ventilatory difficulties. 5. b and c. | ChatGPT: 3/41 students (7.3%) | p = 0.88 |
|  | Lecture: 4/39 students (10.2%) |  |
|  | ChatGPT + Lecture: 4/41 students  (9.7%) |  |
|  |  |  |
| **QUESTION 25.** Absolute contraindications to laparoscopic adrenalectomy include: | | |
| 1. significant abdominal adhesions. 2. adrenal mass greater than 12 cm in size. 3. **invasive adrenal cortical carcinoma with thrombus in the inferior vena cava.** 4. malignant pheochromocytoma. 5. all of the above. | ChatGPT: 5/41 students (12.2%) | p = 0.07 |
|  | Lecture: 4/39 students (10.2%) |  |
|  | ChatGPT + Lecture: 0/41 students  (0%) |  |
|  |  |  |

| **Clinical cases** | | |
| --- | --- | --- |
| **QUESTION 26.** A 60-year-old woman is noted to have a 4-cm left adrenal mass on an abdominal CT scan. The endocrine workup is negative, and the mass is excised laparoscopically. The pathology is reported as a myolipoma. The next step in management is: | | |
| 1. **no additional therapy is indicated because this is a benign tumor.** 2. ask the pathologist to grade the tumor. 3. the patient should receive mitotane. 4. the patient should be followed carefully for development of hypertension. 5. a metaiodobenzylguanidine (MIBG) scan should be obtained. | ChatGPT: 14/41 students (34.1%) | p = 0.09 |
|  | Lecture: 21/39 students (53.8%) |  |
|  | ChatGPT + Lecture: 23/41 students  (56.1%) |  |
|  |  |  |
| **QUESTION 27**. During a right adrenalectomy, severe bleeding is encountered. The possible causes include all of the following  EXCEPT: | | |
| 1. right adrenal vein avulsion at the origin on the inferior vena cava. 2. avulsion of the right hepatic vein branch. 3. disruption of the adrenal capsule. 4. **inadvertent ligation of the upper pole renal artery.** 5. all of the above. | ChatGPT: 3/41 students (7.3%) | p = 0.28 |
|  | Lecture: 3/39 students (7.7%) |  |
|  | ChatGPT + Lecture: 7/41 students  (17.1%) |  |
|  |  |  |
| **QUESTION 28.** A 58-year-old woman has been diagnosed with primary aldosteronism based on appropriate screening and confirmatory testing. A CT scan of the abdomen reveals a 1.0-cm left adrenal mass. Adrenal vein sampling is performed with the results outlined below. Results of adrenal vein sampling:  Right cortisol gradient 2.7:1  Left cortisol gradient 3.4:1  Aldosterone ratio (left:right) 2.5:1  The next step in management would be: | | |
| 1. repeat adrenal vein sampling with ACTH stimulation. 2. **counseling for left adrenalectomy.** 3. counseling for right adrenalectomy. 4. initiation of medical management based on diagnosis of bilateral adrenal hyperplasia. 5. 131I-iodomethyl-norcholesterol (NP-59) scintigraphy to confirm lateralization. | ChatGPT: 8/41 students (19.5%) | p = 0.43 |
|  | Lecture: 6/39 students (15.4%) |  |
|  | ChatGPT + Lecture: 11/41 students  (26.8%) |  |
|  |  |  |
| **QUESTION 29. A 57-year-old male with no significant medical problems presented with a right-sided abdominal mass. Computed tomographic (CT) imaging showed an 18-cm right adrenal tumor with invasion of the upper pole of the right kidney and tumor thrombus extending into the retrohepatic inferior vena cava. Which is the best surgical approach for this patient?** | | |
| 1. Open lumbodorsal posterior approach 2. Open anterior transabdominal approach 3. **Open thoracoabdominal approach** 4. Laparoscopic transperitoneal approach 5. Robot-assisted laparoscopic transperitoneal approach | ChatGPT: 2/41 students (5%) | p = 0.19 |
|  | Lecture: 1/39 students (2.6%) |  |
|  | ChatGPT + Lecture: 5/41 students  (12.2%) |  |
|  |  |  |
| **QUESTION 30.** Which class of antihypertensives is contraindicated during the evaluation of primary aldosteronism? | | |
| 1. Calcium channel blockers 2. Alpha blockers 3. Beta blockers 4. **Aldosterone-receptor blockers** 5. Angiotensin-converting enzyme inhibitors | ChatGPT: 8/41 students (19.5%) | p = 0.55 |
|  | Lecture: 5/39 students (12.8%) |  |
|  | ChatGPT + Lecture: 9/41 students  (22%) |  |
|  |  |  |
